# Neglecting the care of people with schizophrenia: here we go again

**DOI:** 10.1017/S0033291723000247

**Published:** 2023-03

**Authors:** Anthony J. Pelosi, Vijay Arulnathan

**Affiliations:** 1 Priory Hospital, Glasgow G41 3DW, UK; 2 Whyteman's Brae Hospital, Kirkcaldy KY1 2NA, UK

**Keywords:** Community care, discharge pathways, early intervention for psychosis, psychosis, schizophrenia

## Abstract

Specialist early intervention teams consider clinician–patient engagement and continuity of care to be a driving philosophy behind the treatment they provide to people who have developed schizophrenia or a related psychotic illness. In almost all countries where this service model has been implemented there is a dearth of available data about what is happening to patients following time-limited treatment. Information on discharge pathways in England indicates that some early intervention specialists are discharging most of their patients from all psychiatric services after only 2 or 3 years of input. Some ex-patients will be living in a state of torment and neglect due to an untreated psychosis. In the UK, general practitioners should refuse to accept these discharge pathways for patients with insight-impairing mental illnesses.

During the last two decades, specialised early intervention teams have become a core part of psychiatric services in Australia, Canada and the UK. Their vaunted success has influenced policymakers around the world, leading to widespread roll-out of this model of care (Asian Network of Early Psychosis Writing Group, 2012; Cocchi et al., 2018; Csillag et al., 2016; Roe et al., 2021; Stefanis, 2022). These teams provide ‘the gold standard treatment for first-episode psychosis’ and are considered the ‘Jewel in the Crown’ of English psychiatry (French, 2018; Puntis et al., 2020a, 2020b). Their main role has been to treat promptly teenagers and young adults who have newly developed any of the overlapping conditions of schizophrenia, schizoaffective disorder, delusional disorder, affective psychosis, or other non-specified psychotic illness. The teams have protected caseloads to allow time to engage with patients and families and to deliver a range of community-based psychological and social interventions. Antipsychotic medication is used although the dosage of these powerful but dangerous medicines is kept to a minimum. Input is time limited, with an aim to discharge all patients after 3 years.

The rationale rests upon an influential hypothesis that the first 3 years is ‘the critical period’ in psychotic illnesses (Birchwood, Todd, & Jackson, 1998). This predicts that high-quality input during these initial years will lead to better outcomes and, for some patients, will even bring about full recovery (Jackson et al., 2019). There have been concerns that improved outcomes may not be maintained following discharge or transfer of care (Allison, Bastiampillai, Malhi, & Castle, 2019; Bosanac, Patton, & Castle, 2010; Gafoor et al., 2010; Gallagher et al., 2022; Hyatt, Hasler, & Wilner, 2022; Jones et al., 2020; McGorry, Ratheesh, & O'Donoghue, 2018; Pelosi & Birchwood, 2003; Puntis et al., 2020a) and that ‘the duration of treatment that [specialised early intervention] teams offer to patients is not sufficiently long enough to consolidate the therapeutic gains made during treatment…’ (Puntis et al., 2020a). These issues have been examined in recently published Cochrane reviews of international randomised controlled trials (RCTs) and in an important observational study of psychiatric services in Oxfordshire, England (Puntis et al., 2020a, 2020b; Puntis, Oke, & Lennox, 2018).

## Specialised early intervention *v*. treatment as usual

The first Cochrane meta-analysis included three RCTs (from Denmark, Norway and England) and one cluster-RCT (from the USA) with treatment duration of 18 or 24 months. Those treated by the specialised teams had fewer hospital admissions and readmissions and fewer relapses. Hallucinations, delusions and thought disorder were less severe as were the disabling negative symptoms of psychosis such as lack of drive and motivation, blunting of emotions, social withdrawal and poverty of speech and thought. These patients had a better overall quality of life and better general functioning. They reported greater satisfaction with their care. Also, they were more likely to be in education or employment by the end of the study. ‘Recovery’, which involved a combination of symptom stability and improved social attainment, was a co-primary outcome. At the end of treatment in two of the trials, 70% of the specialised care patients were ‘in recovery’ compared with 52% of the controls (Craig et al., 2004; Grawe, Falloon, Widen, & Skogvoll, 2006).

The other specified primary outcome was disengagement from psychiatric services. Across three studies, 24 out of 344 (7%) patients disengaged from the specialised services; 43 of 286 (15%) disengaged from treatment as usual. The Cochrane Collaboration team considered this to be their most confident and most important finding (Puntis et al., 2020b).

This conservative meta-analysis supports Correll et al.'s earlier systematic review of 10 heterogeneous RCTs (from Denmark, England, Hong Kong, Italy, Mexico, Spain and the USA) involving more than 2000 participants. There were ‘small-to-medium’ therapeutic gains across the board in clinical and social domains with specialised early intervention (Correll et al., 2018). The co-primary outcome of psychiatric hospital admission showed that 32% of the specialised care patients were hospitalised at least once compared with 42% of the control participants. Disengagement from psychiatric care was, once again, a primary outcome and favoured the specialised teams; in the combined studies, 21% discontinued treatment compared with 31% of those randomised to treatment as usual (Correll et al., 2018).

## Extended specialised early intervention *v*. specialised early intervention + treatment as usual

In the second Cochrane meta-analysis, extended specialised intervention lasting for a total of 3 years in a trial in Hong Kong (160 participants) and for 5 years in trials in Canada and Denmark (220 and 400 participants) was compared with 2 years of early intervention followed by treatment as usual (Puntis et al., 2020a). Clinically and statistically significant differences did not emerge in measures of hallucinations, delusions, thought disorder, negative symptoms, depressed mood, general functioning and education or employment status. However, satisfaction with care which was assessed only in the Danish trial (Albert et al., 2017) was substantially greater with the extended treatment package. Symptom remission was used as a proxy for the co-primary outcome of ‘recovery’. The RCT in Hong Kong found 78% in remission following extended specialised care compared with 68% of those who received specialised care only for the standard 2 years (Chang et al., 2015). The other two trials were less encouraging. In the Canadian study, 43% were in remission following 5 years of specialised care compared with 38% of those who received 2 years of specialised then 3 years of ‘regular’ care (Malla et al., 2017). A disappointing 22.3% *v*. 21.6% were in remission at the end of the 5-year long Danish trial (Albert et al., 2017).

The other primary outcome was disengagement from treatment. Yet again, this was the most clear and confident finding. Across two of the studies (Chang et al., 2015; Malla et al., 2017), 28 of 192 (15%) patients who received extended specialist care left the trials early compared with 63 of 188 (34%) control patients (Puntis et al., 2020a). At the end of the Danish trial, 90% were in contact with the extended early intervention teams compared with only 56% in contact with services if they had been moved on to treatment as usual (Albert et al., 2017).

It is not surprising that disengagement from treatment has been used as a pre-specified primary outcome. Early intervention specialists have proposed that ‘…the success of EI services is in part due to their emphasis on establishing and maintaining an individual's engagement with the service’ and they consider this a ‘driving philosophy’ of their approach (Birchwood, 2014; Kim et al., 2019; Tindall, Simmons, Allott, & Hamilton, 2018). In clinical practice, every effort is made to prevent disengagement. For example, in Norfolk, Suffolk and Cambridgeshire in the East of England,

‘[p]articipants were considered as having disengaged with services after all possible ways to engage them had been explored by the clinical team. These included: appointment letters, phone calls, text messages, emails, home visits and contact with family, friends and other health, education and social care providers. This process involved several attempts (usually at least six to eight attempts over a 2–3 month period)’ (Solmi et al., 2018).

There has been splendid qualitative and quantitative research on factors that may predict disengagement (Doyle et al., 2014; Kim et al., 2019; Mascayano et al., 2020, 2021; Polillo et al., 2022; Tindall et al., 2018) and an ambitious cluster-RCT of a team-based motivational intervention to improve engagement is well underway in first-episode psychosis teams around England (Greenwood et al., 2021; ISRCTN registry, 2021). The importance of service engagement for this patient group is summarised well in the trial's plain language summary: ‘at least a quarter of all young people drop out of services in the first 12 months, leading to greater risk of poor health and more long-term service use. Families also struggle more’ (ISRCTN registry, 2021).

## Treatment as usual

As is so often the case in health services research, there has been a lack of proper attention to ‘treatment as usual’. The published trials tend to provide only cursory descriptions. These have been drawn together by Correll et al. in a helpful supplementary table that provides some idea of what well-resourced multidisciplinary teams were compared with (Correll et al., 2018). This was mainly old-fashioned office-based appointments with a psychiatrist and/or a nurse. In the Canadian trial of extended early intervention, step-down was either to psychiatric hospital outpatient services or care from a family physician that was, according to the researchers, ‘of variable quality and intensity’ (Lutgens et al., 2015; Malla et al., 2017). It is worrying that, ‘[w]hile transfer to regular care was started within two weeks of randomization, it was dependent on the ability and policies of receiving services and often involved considerable delays (mean 25.7 ± 16.1 weeks)’ (Malla et al., 2017). Another description of treatment as usual, ‘…out-patient medical follow-up with limited community support which focused mainly on crisis intervention’ (Chang et al., 2015), encapsulates perhaps the worst imaginable – and all too common – approach to the management of people with schizophrenia and other psychoses.

## Discharge from specialist early intervention services

Treatment as usual in the two British randomised trials did, at least, involve input from multidisciplinary community mental health teams (Craig et al., 2004; Kuipers, Holloway, Rabe-Hesketh, & Tennakoon, 2004). But this brings us to what has been happening to patients who were under the care of the early intervention for psychosis services in Oxfordshire between 2006 and 2017 (Puntis et al., 2018). The average length of treatment for 701 patients was 1 year and 8 months – even though the stipulated period of involvement was 3 years. No less than 83.5% of the patients were discharged directly to their general practitioners. The remainder were transferred to other psychiatric services.

After a year, about a quarter of those who had been transferred to other psychiatric teams had relapsed; relapses in this group plateaued at close to 50% after 4–5 years. General practitioners referred almost a third of the discharged patients back to psychiatry within 2 years. About 10% of those discharged to primary care are known to have relapsed in the first year with identified relapses plateauing around 15% after about 4 years. Relapse was defined as being admitted to psychiatric hospital or being treated by the crisis resolution and home treatment teams whose role is to head off admission when this would otherwise be required. These episodes could be identified from Oxfordshire's computerised health records system. The problem with this proxy for relapse is that it misses those who deteriorate but do not come to the attention of clinicians. It is a hallmark of psychotic illnesses that insight can be impaired when patients relapse. Symptoms such as paranoid delusions can lead to active avoidance of all assistance. Loss of volition, emotional blunting and poverty of thought reduce the ability to seek help. Based on painful clinical and research experience (Boeing et al., 2007; Murray, Walker, Mitchell, & Pelosi, 1996), we can safely assume that some of these people are sitting at home in a state of quiet neglect listening to hallucinatory voices, with kitchen drawers full of unopened packets of antipsychotic tablets and a relapse management plan that has been untouched since the day they were discharged from assertive community care. Some ex-patients will no longer have a home (Lynch, 2020; Marshall, 1989; Marshall & Gath, 1992).

There is wide variation in the proportion of those who are discharged from all psychiatric services by early intervention teams in England. This has been happening to 47% of patients in Leicester and 55% in Derbyshire (Ahmed, Peters, & Chakraborty, 2019; Phillipson, Akroyd, & Carley, 2014). A recent article reveals that fully three-quarters were discharged by an inner-city London Trust after, on average, 28 months of care (Puntis, Whiting, Pappa, & Lennox, 2021). A conference presentation by leaders in the field from the Birmingham and Solihull National Health Service Trust reported that this happened to 47% of patients in North-West England, 58% in Cornwall, 74% in Norfolk and 77% in Cambridge. The Birmingham and Solihull early intervention services were discharging a third of patients directly to primary care but the theme of their rather jaunty lecture was that they ‘must do better’ and increase this number (Turner & Rainey, undated).

We have sought advice from colleagues working in Canada and Australia and have searched journal articles, book chapters and the ‘grey’ literature but there seems to be no equivalent available information about discharge practices in other parts of the world. We have also raised questions about this issue at online international and national conferences. Sorry to say, but there is an unfathomable complacency amongst British early intervention specialists about the fate of their erstwhile patients. We have not picked up such an attitude amongst clinicians and researchers from other countries and this is reflected in some of the published qualitative research.

Due to gaps in routine data collection, patients' discharge destinations were not documented for the recent detailed evaluation of early psychosis youth services (called headspace) around Australia (Ernst & Young Australia, 2020). However the qualitative findings are telling. Half of those patients who were aware that they were soon to be discharged ‘expressed great concern about what support would be available post discharge’.

One parent was not quite able to put all her concerns into words:

‘…once she leaves the program I want, I want to know where she should go…for some sort of guidance, you know, for the future…Yeah at the moment she went to headspace, headspace is looking after her, but you know, when this program finishes she has to go somewhere else, because I believe that there's not going to umm, it is a long-term thing so there will be some sort of ongoing support required…Yeah. That is the worry I have, once she finishes headspace, what do we do’ (Ernst & Young Australia, 2020).

Another stated:

‘That would be really critical and important for us and our peace of mind because at the moment she's looked after well by headspace, but suddenly that support goes, you know we have got to have some backup.’

A patient summed up their fears as follows:

‘I'm just scared for the future because I'm 25 now, so not going to be with headspace…So I haven't actually talked to anyone about what I'm going to do’ (Ernst & Young Australia, 2020).

Specialist teams in North America are having to navigate the complexities in care pathways that come with various mixes of public and private health care provision and separate funding of early intervention services. One group in Halifax, Nova Scotia are discharging about a third of their patients to primary care (Tibbo, 2022). They provide ongoing support to the receiving general practitioners and offer informal phone contacts to discharged patients about whom they are worried; they are understandably anxious about the adequacy of such an arrangement (Tibbo, 2022). Services in New Haven, Connecticut have reported on several cycles of a quality improvement project to improve ‘this first critical hand off in care’(Gallagher et al., 2022). They describe good liaison with receiving clinicians. Their ongoing Plan-Do-Study-Act cycles have led, so far, to a reduction from 51% to 26% in the proportion of patients whose 3-month care status is unknown.

Jones et al. (2020) have interviewed clinicians, administrators and patients from early intervention services [which they call coordinated specialty care (CSC)] at 36 sites around the USA. They found ‘tremendous variability of discharge practices and policies…’. There were examples of excellent practice. For example, a handful of sites could provide stepped down care from patients' previous therapists and prescribers. One site had ‘clinically oriented follow-up to ensure (or assess) the success of the transition’. However, individuals who were poised to be discharged from some of the programmes expressed concerns about the fixed time limits on their participation. One of the themes that emerged from patient interviews was that,

‘..they appeared to view discharge from CSC as entailing the permanent loss of access to close therapeutic relationships, whether because of cost, access, or standards of care’ (Jones et al., 2020).

Providers shared these concerns. In some areas, it was not possible to access ongoing care from psychiatrists who were comfortable treating psychosis and there could be difficulties ensuring continuing prescription of long-acting injectable antipsychotics and even clozapine.

These researchers provide worrying quotes from professionals and patients that nicely illustrate the theme of inadequate follow-up once separately funded CSC came to an end. Staff at one site,

‘…did kind of an informal calling back to say like, ‘Hey, how are you doing? What's happening?’ and almost nobody had continued in outpatient care. It's a handful, maybe two or three [out of 20 successfully contacted]. And those were the ones with more active family members. They expressed several things. One, that none of the programs they went to were ever like [CSC program] and they wanted to come back to [CSC]…And more than 50% have already dropped out of the care that they had been connected to: they couldn't get the appointments. So, the desire to get care was there, but they were dissatisfied with what they were able to get.’

The frustration of some clinicians emerged clearly:

‘Someone could be in this program functioning beautifully…but then once they leave…they lose it. Suddenly they think they don't need meds anymore…and no one's there to catch them…We've had a lot of really heartbreaking cases like that’ (Jones et al., 2020).

## Here we go again

The approach of time-limited resource intensive intervention followed by rapid stepdown of care has parallels with how people with schizophrenia and related psychotic illnesses were treated in the heyday of asylum care. In the UK, there was decent enough multidisciplinary care in acute admissions wards. But those who did not then remain settled with psychiatric outpatient and general practitioner appointments would end up being ‘warehoused’ in horrible and sometimes disgusting back wards of mental hospitals (Pelosi, 1993). Things became even worse when moves began towards care in the community – and nobody had a clue how to deliver this (Lancet, 1985; Murphy, 1991; Ritchie, Dick, & Lingham, 1994). Elaine Murphy's *After the Asylums*, published in 1991, had a chapter entitled ‘The Disaster Years, 1962–1990’. Referring to people who had been hospitalised for years, she wrote:

‘[w]e do know…that many of the arrangements made when patients first left hospital did not last longer than a few years. Circumstances change, relatives die, relationships do not work out as expected. Some who were discharged mentally well had further breakdowns. Many left their original discharge addresses and drifted into the large towns to live in lodging-houses and cheap hotels, seeking isolation and anonymity, persistently plagued by delusional ideas and troublesome hallucinatory voices. Some ended up in prison. No doubt there were many successes too but similarly we know almost nothing about them’ (Murphy, 1991).

It is difficult to envisage how similar scenarios in countries throughout the world could be prevented by a brief period of treatment from clinicians who specialise solely in the early stages of severe and frequently enduring mental disorders.

## Ongoing care in the community

Many patients who have gone through an episode of psychosis will do well. However, others will have a prolonged and severe and sometimes a devastating illness. It is not possible accurately to predict which patients these are. We are reluctant to give advice on how to provide proper ongoing care to these people in countries where we lack knowledge and experience of their health systems. Commenting on the findings of Jones et al. (2020), Canadian clinician researchers have written an article entitled ‘Moving from islands of order to a sea of chaos: Transitions out of early intervention services for psychosis’ (McIlwaine, Fuhrer, & Shah, 2020). They make practical and deliverable suggestions on improving transitions that could be relevant to countries around the world and not just in North America. However,

‘[a]lthough innovations such as extending [early intervention for psychosis] or ‘stepped-down’ transitional approaches may help to facilitate smoother transfers between services, these strategies are far from solving the larger health care problem: that post-[early intervention for psychosis] mental health care is often ‘the opposite of recovery,’ expensive, inaccessible, agnostic to client needs, and thus a potential contributor to disengagement. Unfortunately, these issues coincide with other treatment, financial, and structural barriers encountered by those who could benefit from receiving psychopharmacologic or supportive, therapeutic care – barriers that themselves warrant further research to identify possible solutions to mitigate their negative effects and improve mental health care services in general’ (McIlwaine et al., 2020).

Nobody, we hope, will disagree with those views nor with Jones et al. (2020) when they argue that,

‘…. [coordinated specialty care] represents a level of quality that should in fact characterize mental health services in general, as underscored by both staff and client concerns regarding the values and offerings of postdischarge services.’

We are able to give advice on how these transitions should be tackled in the UK – where there can be no excuse for any patient with a known psychotic illness to be discharged to a ‘sea of chaos’. No matter how stretched they are, general adult community mental health teams in Britain should insist on taking over the management of essentially every person who has had their couple of years (or less) of ‘gold standard treatment’ (Kirkbride et al., 2017; Lester et al., 2012; Puntis et al., 2020a, 2021). These teams will be faced with yet more difficult decisions about the rationing of clinical resources. But given that 10% of discharged patients in Oxfordshire are known to have relapsed within a year and a third were referred back to psychiatric services within 2 years, it is likely that community mental health teams would save themselves time and would spare many patients and families from heartache if they routinely took on ‘this first critical hand off in care’. This will allow the establishment of coordinated, individualised and needs-based community care plans – and contingency plans – that are appropriate for people with insight-impairing illnesses and that can be sustained during good times and bad over the ensuing years and decades.

Should such patients be discharged from psychiatric services immediately following a brief period of intensive input, family doctors should take control. They should write to the consultant psychiatrist in the early intervention team agreeing, as always, to shared care of their patient but refusing to accept the discharge plan. Copy letters should be sent to the catchment area consultant general psychiatrist and the medical director of the local National Health Service Trust. This letter should point out that the proposed care pathway flies in the face of clinical experience, all the research evidence, and the findings of thousands of inquiries into critical incidents and community care tragedies (Reith, 1998; Ritchie et al., 1994).

Early intervention treatment programmes around the world have been based upon on a hypothesis that the first 3 years of illness is *the* critical period in the course of schizophrenia and other psychotic illnesses (Birchwood et al., 1998). This is and always was a ridiculous hypothesis. The early months and years after onset are, of course, *a* critical period. But everybody knows that people with schizophrenia – and schizoaffective disorder and manic-depressive psychosis and delusional disorder and other psychoses – may encounter critical times throughout their illness journey. Well-resourced, well-coordinated multidisciplinary treatment from specialist mental health clinicians is required during each and every one of these periods, with continuing care that will help to prevent them.

## Conclusion

Over the past 25 years, early intervention specialists have used modern public relations techniques and brilliant political skills to influence health care policy around the world (Allison et al., 2019; Bertolote & McGorry, 2005; Csillag et al., 2016; House of Commons, 2021; McGorry, 2015; Pelosi & Birchwood, 2003; Stefanis, 2022; The President's Office Republic of Maldives, 2022). But a short-term approach to psychiatry combined with self-imposed lack of clinical experience has meant that many of these clinicians have no understanding of the realities of providing care to patients with major mental disorders – for as long and intensively as required. They have closed their eyes and minds to the ongoing needs of their former patients and have somehow failed to notice one of the most worrying findings ever to emerge from mental health services research. In numerous areas across the UK, the most important factor in disengagement of people with insight-impairing illnesses from psychiatric care is planned and deliberate discharge by early intervention for psychosis teams (Ahmed et al., 2019; London Early Intervention in Psychosis Clinical Reference Group, 2016; Phillipson et al., 2014; Puntis et al., 2018, 2021; Turner & Rainey, undated). This may also be the case in Australia, Canada and the USA and, we fear, in other countries where well-intentioned policymakers have been influenced by the early intervention movement. Family doctors, general psychiatry teams, health service managers, advocacy organisations, politicians and patients and their families should do everything they can to put a stop to this unacceptable practice. Psychiatry can and really must do better.
